# Overexpression of CCN3 Inhibits Inflammation and Progression of Atherosclerosis in Apolipoprotein E-Deficient Mice

**DOI:** 10.1371/journal.pone.0094912

**Published:** 2014-04-10

**Authors:** Jun Liu, Yingang Ren, Li Kang, Lihua Zhang

**Affiliations:** Department of Geriatrics, Tangdu Hospital, The Fourth Military Medical University, Xi'an, China; University of Pittsburgh School of Medicine, United States of America

## Abstract

**Background:**

Cysteine-rich 61/connective tissue growth factor/nephroblastoma overexpressed (CCN) 3 has been recently reported to play a role in regulating inflammation of vascular endothelial cells. However, the role of CCN3 in atherosclerosis, which is characterized by vascular inflammation, remains unclear.

**Hypothesis and Objectives:**

Overexpression of CCN3 may relieve the inflammation response in and inhibit the progress of atherosclerosis. We aimed to explore the potential roles of CCN3 in inflammation in atherosclerosis.

**Strategy and Main Results:**

In in vitro studies using cultured human aortic endothelial cells and human umbilical vein endothelial cells, CCN3 mRNA and protein expression significantly decreased in response to tumor necrosis factor-α and interleukin-1β treatments (p<0.05), when analyzed by quantitative real-time polymerase chain reaction and Western blot. Using a mouse model of atherosclerosis, the mRNA and protein levels of CCN3 decreased by 72.2% (p = 0.041) and 86.4% (p = 0.036), respectively, compared with levels in wild-type control mice, respectively. Overexpression of CCN3 by adenovirus-mediated gene overexpression decreased low-density lipoprotein cholesterol by 48.9% (p = 0.017), total cholesterol by 58.9% (p = 0.031), and triglycerides by 56.8% (p = 0.022), and it increased high-density lipoprotein cholesterol level by 2.16-fold (p = 0.039), compared with control groups. Additionally, a reduced plaque area and increased fibrous cap were observed (p<0.05). Furthermore, CCN3 overexpression decreased cell adhesion molecule-1 mRNA expression by 84.7% (p = 0.007) and intercellular adhesion molecule-1 mRNA expression by 61.2% (p = 0.044). Inflammatory factors, including matrix metalloproteinases, cyclooxygenase 2, and tissue factor also significantly (p<0.05) decreased with CCN3 overexpression in the atherosclerotic mouse model. Additionally, CCN1 and CCN2, which have been reported to be highly expressed in aortic atherosclerotic plaques, were significantly downregulated (p<0.05) by CCN3 overexpression.

**Conclusion:**

CCN3 overexpression is associated with control of inflammatory processes and reversion of dyslipidemia in the process of atherosclerosis, which implies that CCN3 may be a promising target in the treatment of atherosclerosis.

## Introduction

Atherosclerosis is a worldwide disease that induces acute cardio-cerebrovascular events, causing serious damage to human health [1]. Atherosclerosis causes chronic inflammation that is characterized by the accumulation of lipids, fibrous tissue, and inflammatory cells in arteries, where the interactions of various inflammatory factors and cells result in vascular injuries [2]. Elucidating the molecular mechanism in the regulatory inflammation network will provide new insights for the treatment of atherosclerosis.

Recently, a group of secreted extracellular matrix-associated signaling proteins, the cysteine-rich 61/connective tissue growth factor/nephroblastoma overexpressed (CCN) family, has been implicated in regulating chronic inflammatory diseases, such as rheumatoid arthritis, atherosclerosis, neuroinflammatory pathologies, and inflammatory kidney diseases [3]. To date, six members of the family have been characterized, including cysteine-rich protein 61 (CCN1), connective tissue growth factor (CCN2), nephroblastoma overexpressed (CCN3), Wnt-inducible signaling pathway protein 1 (CCN4), Wnt-inducible signaling pathway protein 2 (CCN5), and Wnt-inducible signaling pathway protein 3 (CCN6) [4], [5]. These proteins are proposed to be involved in diverse biological progresses. For example, CCN1 and CCN2 have been demonstrated to play important roles in cell proliferation, migration, and adhesion [6]. CCN proteins have recently been indicated as a new class of modulators in inflammatory processes. Of these, CCN1 and CCN2 have been demonstrated to be highly expressed in arteriosclerotic lesions [7], [8], [9], [10], which indicates that CCN plays a critical role in arteriosclerosis. However, the role of CCN3 in arteriosclerosis is not understood.

CCN3 was first identified in nephroblastoma tissue from newborn chicks infected with the MAV-1 avian retrovirus [11]. CCN3 exhibits wide distribution in diverse tissues, including skeletal and cardiac muscle, nervous system, cartilage, lung, and kidney (reviewed in [12]). For about two decades, the molecule has been demonstrated to participate in tumorigenesis, hematopoiesis, and bone development [6], [13], [14]. More recently, CCN3 has been demonstrated to be capable of attenuating inflammatory pain [15]. CCN3 expression is found in endothelial cells, fibroblasts, and smooth muscle cells in vascular vessels [16], [17]. CCN3 knockout mice have been found to have enhanced neointimal hyperplasia under endothelial injury [18], which implies that CCN3 has an important role in the regulation of atherosclerotic vascular disease. Lately, CCN3 has been reported to be a novel modulator of endothelial inflammation, suggesting that CCN3 might have a potential role in regulating atherosclerosis progress [12]. However, the precise role of CCN3 in atherosclerosis is under-explored.

In the present study, we aimed to investigate the role of CCN3 in atherosclerosis. We found that the overexpression of CCN3 in vivo relieved dysregulated blood lipid metabolism, reduced the plaque area, and increased the fibrous cap, which were beneficial for plaque stability. Furthermore, the overexpression of CCN3 markedly inhibited gene expression of adhesion molecules and inflammatory mediators. Taken as a whole, we identified that CCN3 contributed to the control and coordination of inflammatory processes in atherosclerosis.

## Materials and Methods

### Ethics statement

This study was carried out in strict accordance with the guidelines of the National Health and Medical Research Council for the Care and Use of Animals for Experimental Purposes in China. The protocol was approved by the Institutional Animal Care and Use Committee of the Fourth Military Medical University. All efforts were made to minimize suffering. For euthanasia, mice were injected with pentobarbital sodium (100 mg/kg, Merck KGaA, Darmstadt, Germany) by intraperitoneal injection.

### Animals and cell culture

A total of 90 male apolipoprotein E-deficient mice (ApoE−/−, 3 weeks old, weighing 20–25 g) were purchased from Beijing Biocytogen (Beijing, China). The mice were housed as per standard protocols and fed a high-fat diet (20% fat, 20% sugar, and 1.25% cholesterol) with free access to water. Human aortic endothelial cells (HAECs) and human umbilical vein endothelial cells (HUVECs) obtained from Lonza (Walkersville, MD, USA) were maintained in endothelial basal medium-2 and supplemented with 2% fetal bovine serum (FBS) at 37°C with 5% CO_2_ in an incubator (Life Technologies, Baltimore, MD, USA). Cells were treated with recombinant human tumor necrosis factor (TNF)-α (10 ng/ml) or interleukin (IL)-1β (4 ng/ml) from R&D Systems, Inc. (Minneapolis, MN, USA), respectively, and incubated for 24 h. Cells treated with phosphate buffered saline (PBS) were taken as control. Total cell RNA or protein was harvested according standard protocols for further analysis.

### Adenovirus construction and transfection

All recombinant adenovirus were constructed as previously described [19]. Briefly, full-length CCN3 cDNA was amplified and subcloned into pAdTrack-cytomegalovirus (CMV), an adenoviral shuttle plasmid, whereas green fluorescent protein (GFP) was used as a non-specific control. Then, the recombinant shuttle plasmids pAdTrack-CMV and pAdEasy-1 were homologously recombined in *Escherichia coli* strain BJ5183. The obtained recombinant plasmids were transfected into 293 cells to generate recombinant adenovirus. The virus was amplified and purified, and titers were determined using the p24 ELISA kit (Cell Biolabs, Inc., San Diego, CA, USA).

Atherosclerotic lesions were induced by using the carotid-artery intubation technique [20]. Typically, the collars were removed eight weeks after surgery for subsequent experiments. The mice were randomly grouped into the control group, which received a 200-μl PBS injection; the Ad-GFP group, which received a 200-μl (1×10^10^ plaque-forming units of virus) injection of recombinant adenovirus expressing GFP; and the Ad-CCN3 group, which received 200-μl (1×10^10^ plaque-forming units) injection of recombinant adenovirus expressing CCN3. Two weeks later, a second injection was administered in a similar fashion as previously described, and the mice were euthanized for analysis two weeks after the second injection.

### Quantitative real-time polymerase chain reaction (qRT-PCR)

Total RNA was extracted from the right common carotid arteries with the TRIzol reagent (Invitrogen, Carlsbad, CA, USA) and 5 μg of the total RNA was reverse-transcribed into cDNA using M-MLV reverse transcriptase (Clontech Laboratories, Inc., Palo Alto, CA, USA). The cDNAs were used as templates for qRT-PCR. The qRT-PCR mixture contained 5 μl SsoFastTM EvaGreen Supermix (BIO-RAD, Hercules, CA, USA), 1 μl of cDNA (diluted 1∶50), and 2 μl each of the forward and reverse primers (1 μM) to a final volume of 10 μl. The PCR procedure was as follows: 94°C for 4 min; 94°C for 20 s, 55°C for 30 s; 72°C for 20 s; 2 s for plate reading for 35 cycles; and a melting curve from 65 to 95°C. GAPDH was used as a control for normalizing the gene expression. Three independent experiments were performed. The data obtained were calculated by 2^−ΔΔCt^ and treated for statistical analysis as described previously [21], followed by an unpaired sample t-test.

### Western blotting

Proteins were extracted from tissues of the right common carotid arteries and protein concentrations were measured by using the Bradford method. Twenty μg of protein was separated by 12% sodium dodecyl sulfate polyacrylamide gel electrophoresis (SDS-PAGE) followed by electro-blotting onto a nitrocellulose membrane (Amersham, Little Chalfont, UK). Then, the membrane was incubated with 2% nonfat dry milk in Tris-buffered saline to block non-specific binding at room temperature for 1 h. Next, the membrane was incubated with primary antibodies (Santa Cruz, CA, USA) diluted in the blocking buffer overnight at 4°C. Subsequently, the membrane was incubated in horseradish peroxidase–conjugated goat anti-rabbit IgG (Boster Corporation, Wuhan, China) and diluted in the blocking buffer for 1 h. 4-Chloro-1-naphthol (4-CN), a horseradish peroxidase substrate, was used for protein visualization.

### Serum sample analysis

Blood samples were collected via mouse-tail vein bleeding. The serum was isolated by centrifugation. Serum levels of cholesterol, triglyceride, low-density lipoprotein cholesterol (LDL-C), and high-density lipoprotein cholesterol (HDL-C) were measured using the ELISA kit (Banyi, Shanghai, China). Matrix metalloproteinases (MMP) concentrations of MMP-2 and MMP-9 in serum were detected using the ELISA kit (Bluegene, Shanghai, China), as per standard procedures.

### Hematoxylin-eosin (HE) staining for analysis of atherosclerotic plaque

Before carotid artery isolation, the mouse heart was perfused with PBS, followed by 4% paraformaldehyde for 30 min under physiological pressure. Afterwards, the isolated carotid artery was fixed with 4% paraformaldehyde for 12 h, then embedded in paraffin and cut into 5-μm serial sections. Hematoxylin was used for cytoplasm staining. Corresponding sections were stained for 10 min at room temperature. According to standard protocols, sections were then washed with running water, Scott promote blue liquid, and 1% hydrochloric acid alcohol differentiation liquid. Thereafter, sections were stained with 0.5% eosin (Merck, Whitehouse Station, NJ, USA). For lesion area analysis and calculation of the plaque area, fibrous cap, cap-to-core ratio, and intima-media thickness, sections were observed using Image-Pro Plus 5.0 software (Media Cybernetics, Bethesda, MD, USA).

### Statistical analysis

Data were expressed as the mean ± standard deviation. The statistical significance was determined by using the Student t test for differences between two groups and one-way ANOVA for differences among multiple groups. Error bars were expressed as standard deviation. A p value of less than 0.05 was considered statistically significant. All statistical analyses were performed using SPSS version 11.5 (SPSS Inc., Chicago, IL, USA).

## Results

### Proinflammatory stimuli inhibited CCN3 expression *in vitro*

**Figure 1:**
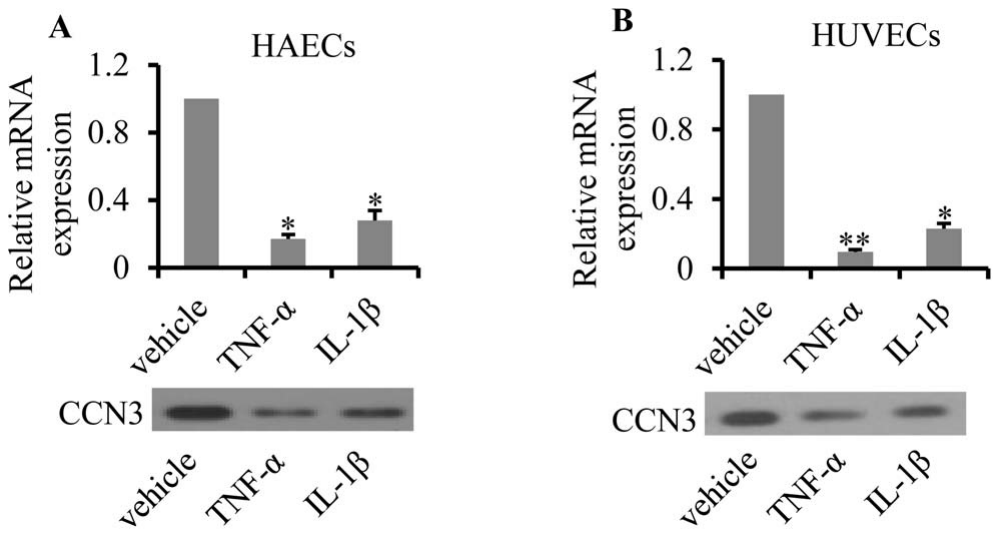
Effect of proinflammatory cytokines on CCN3 expression. HAECs (A) and HUVECs (B) were treated with TNF-α (10 ng/ml) or IL-1β (4 ng/ml) for 24 h. Total RNA and cell protein were harvested for qRT-PCR or Western blot analysis, respectively. mRNA expression was normalized to GAPDH (*N* = 6, *p<0.05 and **p<0.01). Protein loading was normalized to equal amounts of cells.

To investigate the effect of proinflammatory stimuli on CCN3, we investigated CCN3 levels in cultured HAECs and HUVECs after TNF-α and IL-1β challenges. Compared with control groups, CCN3 mRNA expression was significantly decreased by 83% (p = 0.027) and 72% (p = 0.042) in HEAC cells in response to TNF-α and IL-1β treatments, respectively (Fig. 1A upper). Similar results were obtained in HUVECs, where CCN3 mRNA expression was significantly decreased by 89.9% (p = 0.009) and 77.1% (p = 0.017) in response to TNF-α and IL-1β treatments, respectively (Fig. 1B upper). The results were confirmed by Western blot analysis (Fig. 1A–B lower).

### Expression of CCN3 was reduced in atherosclerosis

**Figure 2:**
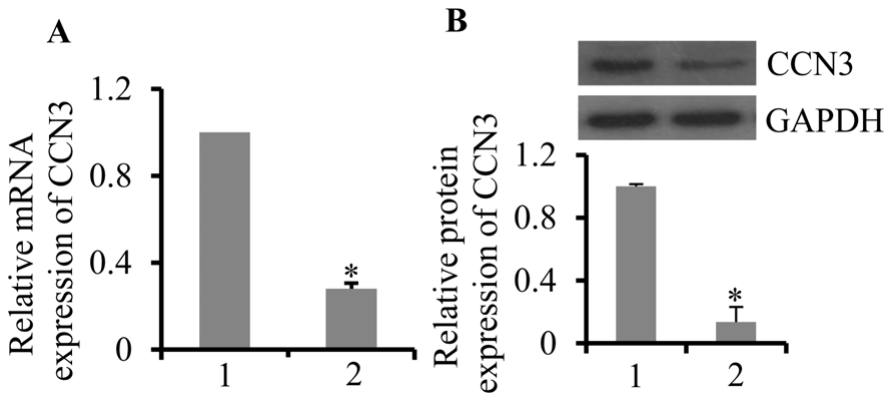
Expression of CCN3 in atherosclerosis. The mRNA level (A) and protein level (B) of CCN3 in the left common carotid artery of mice were evaluated by qRT-PCR and Western blot analysis, respectively. 1 represents control mice (wild-type C57BL; 6 mice); 2 represents 25-week-old ApoE^−/−^ mice fed a high-fat diet. GAPDH was used as a control. *p<0.05, *N* = 30 per group.

Whether CCN3 expression is altered in atherosclerosis is poorly understood. CCN3 expression levels in the left common carotid artery in a mouse model of atherosclerosis were analyzed. The results showed that mRNA levels of CCN3 in the left common carotid artery was reduced by 72.2% (p = 0.041) and protein levels were reduced by 86.4% (p = 0.036) in ApoE^−/−^ mice, compared with those of the control wild-type mice (Fig. 2A–B). These results implied that CCN3 expression was inhibited in atherosclerosis progression.

### Overexpression of CCN3 alleviated atherosclerosis

**Figure 3:**
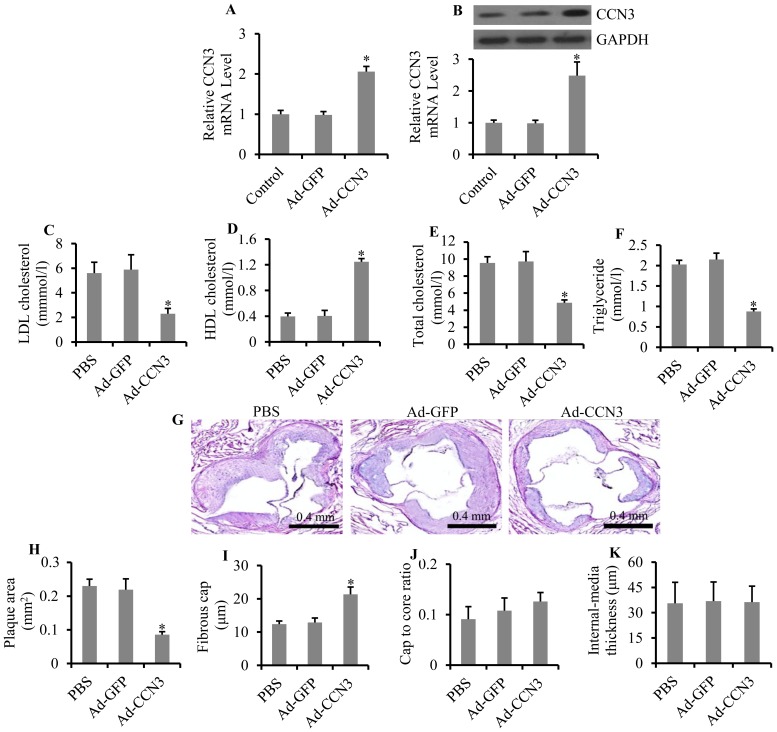
Effect of overexpression of CCN3 on atherosclerosis. CCN3 mRNA (A) and protein (B) levels in different treatment groups of mice were analyzed by qRT-PCR and Western blot, respectively. PBS, mice injected with PBS as a control; Ad-GFP, mice injected with recombinant adenovirus expressing GFP as a non-specific control; Ad-CCN3, mice injected with recombinant adenovirus expressing CCN3. Serum levels of LDL cholesterol (C), HDL cholesterol (D), total cholesterol (E), and triglycerides (F) in the three groups of mice are shown. Cross-section histological analyses of plaques stained by HE (G) are shown. Quantitative analysis of plaque area (H), fibrous cap (I), cap-to-core ratio (J), and intima-media thickness (K) in the three groups of mice are also shown. *p<0.05 vs. PBS or Ad-GFP denotes a statistically significant difference.

To test whether overexpression of CCN3 alleviates atherosclerosis, we constructed recombinant adenovirus expressing CCN3, for *in vivo* injection. The expression of CCN3 in tissues of the left common carotid artery was analyzed. The results showed that CCN3 mRNA expression was increased by 2.48-fold (p = 0.028) and protein expression was increased by 2.06-fold (p = 0.017) after injection of recombinant adenovirus expressing CCN3 (Fig. 3A–B). At the end of the experiment, we measured the body weight and organ weight, including heart, liver, spleen, and kidney, in different groups. Results showed that body, liver, and kidney weights were markedly increased in the Ad-CCN3 transfected group, while heart and spleen weights remained constant among all groups (Table 1). The body weight gain was 16.7% (p = 0.023), the liver weight gain was 14.9% (p = 0.047), and the kidney weight gain was 3% (p = 0.031). To further explore the effect of CCN3 overexpression on atherosclerosis, serum levels of LDL-C, HDL-C, total cholesterol, and triglycerides were measured. The overexpression of CCN3 significantly decreased LDL cholesterol by 48.9% (p = 0.017), total cholesterol by 58.9% (p = 0.031), and triglyceride by 56.8% (p = 0.022), and it increased HDL cholesterol level by 2.16-fold (p = 0.039), compared with mice that received PBS or recombinant adenovirus expressing GFP (Fig. 3C–F).

**Table 1:** Body and organ weights of animals.

Groups	n	BW (g)	HW (g)	LW (g)	SW (g)	KW (g)
PBS	30	33.2±2.8	0.184±0.051	0.841±0.078	0.096±0.002	0.524±0.016
Ad-GFP	30	33.9±3.5	0.191±0.093	0.835±0.066	0.111±0.007	0.539±0.021
Ad-CCN3	30	38.7±2.7*	0.187±0.075	0.967±0.083*	0.105±0.003	0.583±0.018*

*versus PBS or Ad-GFP group denotes p<0.05. BW, body weight; HW, heart weight; LW, liver weight; SW, spleen weight; KW, kidney weight.

Additionally, we evaluated the effect of overexpression of CCN3 on plaque stability (Fig. 3G). Ad-CCN3-infected mice demonstrated a significantly decreased (62.6%, p = 0.043) plaque area, compared with PBS-injected or Ad-GFP-infected mice (0.0857±0.09 mm^2^ vs. 0.230±0.021 mm^2^ or 0.219±0.032 mm^2^, respectively; Fig. 3H). The fibrous caps in the Ad-CCN3-infected group (21.36±2.02 μm) showed a remarkable increase (72.9%, p = 0.036), compared with the PBS (12.35±0.975 μm) or Ad-GFP groups (12.85±1.356 μm; Fig. 3I). However, the cap-to-core ratio and the intima-media thickness showed no significant differences among the three groups (Fig. 3J–K).

### Overexpression of CCN3 suppressed gene expression of cell adhesion molecules

**Figure 4:**
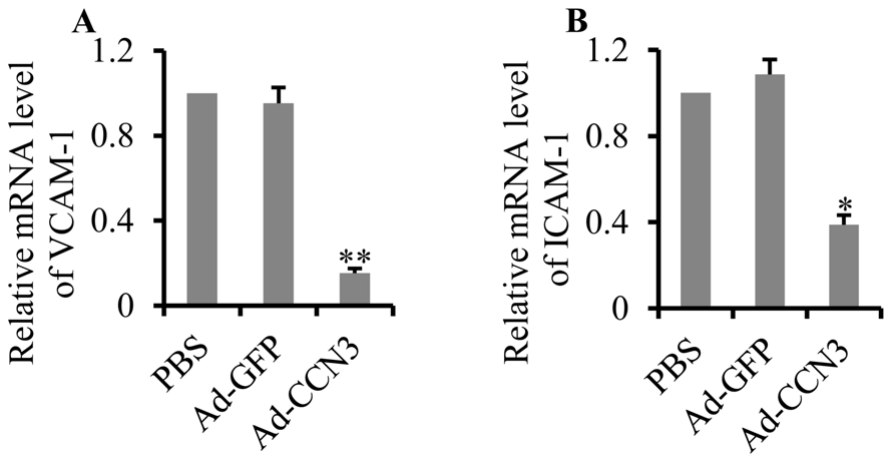
Effect of CCN3 overexpression on gene expression of adhesion molecules. qRT-PCR was used to analyze the mRNA levels of VCAM-1 (A) and ICAM-1 (B) in tissues of the left common carotid artery. *p<0.05 or **p<0.01 vs. PBS or Ad-GFP denotes a statistically significant difference.

One of the earliest events during the initiation of atherosclerosis is the high expression of adhesion molecules by vascular endothelial cells, including vascular cell adhesion molecule-1 (VCAM-1) and intercellular adhesion molecule-1 (ICAM-1) [22], [23]. We aimed to examine whether the overexpression of CCN3 in vivo has the same effect on VCAM-1 and ICAM-1 gene expression. mRNA levels of VCAM-1 and ICAM-1 in the left common carotid artery were determined by qRT-PCR. The results showed that the overexpression of CCN3 markedly decreased VCAM-1 mRNA expression by 84.7% (p = 0.007; Fig. 4A) and ICAM-1 mRNA expression by 61.2% (p = 0.044; Fig. 4B) in the Ad-CCN3 group, while the PBS and Ad-GFP groups showed high abundances of VCAM-1 and ICAM-1 mRNA.

### Overexpression of CCN3 relieved inflammatory response in atherosclerosis

**Figure 5:**
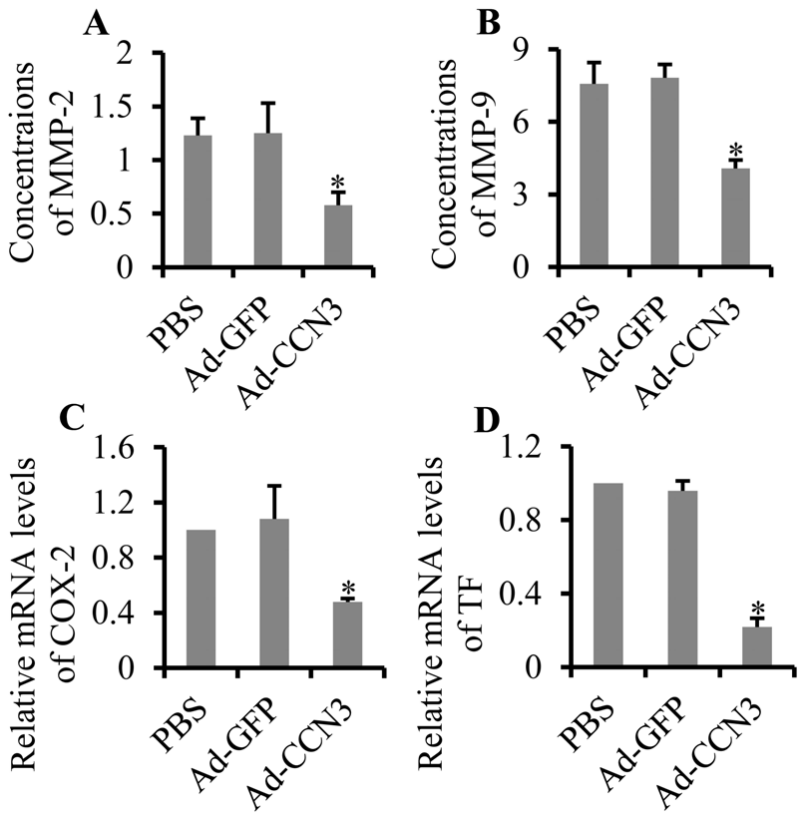
Effect of CCN3 overexpression on inflammation in atherosclerosis. The concentrations of MMP-2 (A) and MMP-9 (B) in serum were measured by ELISA. Gene expression of COX-2 (C) and TF (D) in tissues of the left common carotid artery was analyzed by qRT-PCR. *p<0.05 vs. PBS or Ad-GFP denotes a statistically significant difference.

High expression of inflammatory factors is responsible for the progression of atherosclerosis and plaque instability, including matrix metalloproteinases (MMPs), cyclooxygenase 2 (COX-2), and tissue factor (TF). To investigate whether CCN3 is capable of regulating the inflammatory factors in atherosclerosis, we first analyzed the expression of MMP-2 and MMP-9 by ELISA. The results showed that the overexpression of CCN3 markedly reduced serum concentrations of MMP-2 (53.6%, p = 0.022) in the Ad-CCN3 group, compared with those in the PBS and Ad-GFP groups (0.58±0.12 ng/ml vs. 1.25±0.28 ng/ml and 1.23±0.16 ng/ml, respectively, Fig. 5A). Concentrations of MMP-9 were also significantly reduced (46.3%, p = 0.028) in the Ad-CCN3 group (4.07±0.35 ng/ml), compared with those in the PBS group (7.58±0.87 ng/ml) and in the Ad-GFP group (7.83±0.54 ng/ml; Fig. 5B). Furthermore, gene expression of COX-2 and TF was analyzed by qRT-PCR. The results showed that mRNA levels of COX-2 (Fig. 5C) decreased by 51.6% (p = 0.037) and TF decreased by 77.3% (p = 0.027; Fig. 5D) in atherosclerotic lesions of the left common carotid artery by overexpression of CCN3. These results showed that CCN3 inhibited the inflammatory response in atherosclerosis.

### Overexpression of CCN3 had a negative effect on gene expression of CCN1 and CCN2

**Figure 6:**
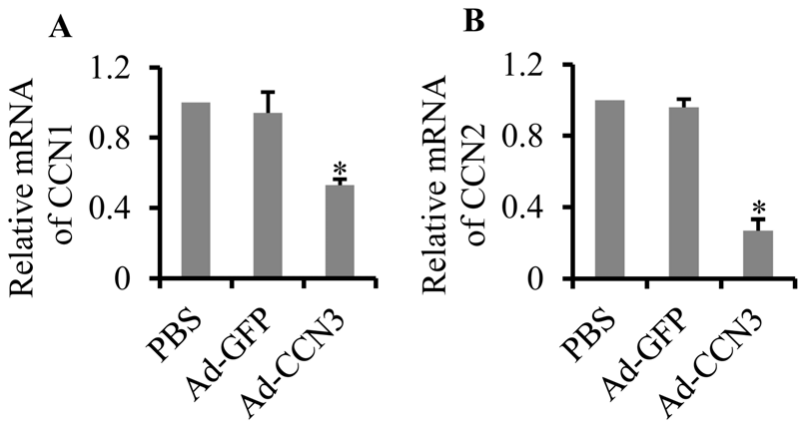
Effect of CCN3 overexpression on CCN1 and CCN2 expression. The mRNA levels of CCN1 (A) and CCN2 (B) in tissues of the left common carotid artery were analyzed by qRT-PCR. *p<0.05 vs. PBS or Ad-GFP denotes a statistically significant difference.

CCN1 and CCN2 have been reported to be highly expressed in aortic atherosclerotic plaques of a mouse model of atherosclerosis and human atherosclerotic lesions [7], [8], [9], [10]. Additionally, CCN3 expression is opposite to CCN1 and CCN2 expression. We aimed to explore the effect of the overexpression of CCN3 on CCN1 and CCN2 levels in atherosclerosis. The results showed that the overexpression of CCN3 significantly reduced gene expression of CCN1 (by 47.1%, p = 0.039) and of CCN2 (by 72.3%, p = 0.027) in atherosclerotic lesions (Fig. 6).

## Discussion

In the present study, we provide evidence that CCN3 is a novel regulator of the inflammatory process of atherosclerosis. The overexpression of CCN3 by adenovirus-mediated gene expression in vivo showed beneficial effects in relieving atherosclerosis. This result implies that CCN3 may have a role in the treatment of atherosclerosis.

CCN3 expression has been found in diverse vessels, including the carotid arteries, ascending aorta, and the thoracic aorta [12]. Laminar shear stress and statins that have anti-inflammatory effects increase CCN3 expression, implying that CCN3 possesses anti-inflammatory functions. Moreover, in response to TNF-α treatment, CCN3 expression is decreased in vascular endothelial cells [12]. In line with these findings, we also demonstrated that CCN3 expression was suppressed in vascular endothelial cells in vitro, in response to TNF-α and IL-1β stimulation. We further showed that CCN3 expression was inhibited in atherosclerosis. We hypothesized that the overexpression of CCN3 might have beneficial effects in improving atherosclerosis. As expected, the overexpression of CCN3 alleviated the dysregulated lipid metabolism by reducing levels of cholesterol, triglycerides, and LDL-C, and by increasing the level of HDL-C in an atherosclerosis mouse model. Furthermore, the plaque area decreased and the fibrous cap increased, which improved the stability of atherosclerotic plaques. Interestingly, CCN3 was revealed to interact with IL-33, which has been suggested to have protective effects against atherosclerosis [24], [25].

One of the earliest important events during the initiation of atherosclerosis is the expression of adhesion molecules by vascular endothelial cells, including VCAM-1 and ICAM-1 [22], [23]. These molecules recruit immune cells, such as monocytes, to the vascular wall. In the vascular wall, these cells mature into macrophages that internalize the lipids and become foam cells, which accelerate the development and progression of atherosclerosis [22], [23]. In the present study, we found that loss of CCN3 was correlated with VCAM-1 and ICAM-1 expression in atherosclerosis, and the overexpression of CCN3 significantly inhibited the expression of these genes. In vascular endothelial cells after TNF-α and IL-1β treatments, a deficiency in CCN3 promoted VCAM-1 and ICAM-1 expression, whereas an overexpression of CCN3 inhibited VCAM-1 and ICAM-1 expression in vitro[12]. Additionally, the underlying mechanism of CCN3 regulation of VCAM-1 is proposed to be through inhibition of the NF-κB pathway. NF-κB has been suggested to modulate endothelial inflammation and atherosclerosis through regulating inflammatory gene expression, such as VCAM-1 [26].

Further experiments demonstrated that the overexpression of CCN3 markedly reduced serum concentrations of MMPs. Increasing evidence has shown that MMPs are hyper-expressed in atherosclerosis and that they substantially degrade the extracellular matrix and reduce plaque stability [27]. Studies have demonstrated that MMP-2 is significantly upregulated in unstable plaques [28], implying that MMP-2-mediated degradation of the extracellular matrix is an important risk factor for unstable plaques. MMP-9 is highly expressed in unstable plaques and in the serum of patients with unstable plaques [29]. CCN3 has also been reported to inhibit inflammatory pain through the regulation of MMP-2 and MMP -9 [15]. TF is a regulator of coagulation and hemostasis that activates the coagulation system when the fibrous cap is broken, resulting in thrombosis [30], [31]. COX-2 was reported to be a proinflammatory factor that is highly abundant in atherosclerotic lesions [32]. The metabolic products of COX-2 promote cell adhesion, platelet aggregation, and contraction and relaxation of vessels, which accelerate the progression of atherosclerosis [32]. Our results demonstrated that the overexpression of CCN3 suppressed gene expression of TF and COX-2. Overall, CCN3 has shown anti-inflammatory effects in atherosclerosis.

Atherosclerosis is defined as a complex inflammatory response characterized by the accumulation of lipid in arteries [33]. Clinical study has demonstrated that high levels of serum LDL and low levels of serum HDL are associated with the pathogenesis of atherosclerosis [34]. Monocytes and macrophages migrate into the intima and use scavenger receptors to swallow modified modified LDL, such as oxidized LDL. These monocytes and macrophages then form into foam cells. These events mark the initial steps in the development of atherosclerosis [35], [36], [37]. Moreover, oxidized LDL stimulates macrophages and vascular endothelial cells to express inflammatory factors, including VCAM-1 and monocyte chemoattractant protein-1 [2], [35], which promote atherosclerosis. Thus, lipemia is highly associated with inflammation during atherosclerosis. In the present study, we found that CCN3 significantly inhibited serum levels of LDL and increased serum levels of HDL, which might contribute to the suppression of inflammation and thus lead to stable plaques. Additionally, we found that overexpression CCN3 significantly increased the body weights in mouse models of atherosclerosis. Results showed that liver and kidney weights were markedly increased, whereas heart and spleen weights remain constant among all groups. Overexpression of CCN3 may improve liver and kidney functions which are deregulated during arteriosclerosis. Weight loss is a typical symptom of inflammatory diseases [38]. Therefore, the data further indicated that CCN3 has an inhibitory effect on inflammation.

CCN3 may have opposite functions to CCN1 and CCN2. Previous studies have shown that CCN1 and CCN2 are highly induced during cell proliferation, while CCN3 is suppressed [39], [40]. In the present study, we observed that the overexpression of CCN3 downregulated gene expression of CCN1 and CCN2, indicating that the three CCN proteins had positive or negative effects in atherosclerosis. Increasing evidence has demonstrated that CCN gene regulation by cytokines is cell-specific or CCN-specific. CCN1 expression is inhibited by TNF-α in chondrocytes, but it is highly induced by proinflammatory cytokines in osteoblasts [41], [42]. TNF-α induces CCN2 in synovial cells, but it suppresses its expression in chondrocytes [43]. TNF-α accelerates chronic pancreatic inflammation by positive regulation of CCN2 expression [44]. TGF-β1 has been shown to be capable of increasing gene expression of CCN1, CCN2, and CCN4, but it inhibits CCN3 expression in cultured astrocytes; TNF-α induces CCN3 expression, but it represses the expression of other CCN genes [3]. Therefore, the ability of cytokines to exert pro- or anti-inflammatory effects might depend on their ability to generate different profiles of CCN gene expression.

Taken as a whole, CCN3 overexpression is associated with control of inflammatory processes and reversion of dyslipidemia. Our data also implied that manipulation of CCN3, such as adenovirus-mediated overexpression of CCN3 in vivo, attenuated the inflammatory progress in atherosclerosis. This suggests that CCN3 is a promising target for atherosclerosis treatment. However, further investigations are warranted to delineate the molecular mechanism of CCN3 in regulating the inflammatory progress in atherosclerosis.
